# Learning Competency Framework and Approach for the Displaced Rohingya Children Living in Bangladesh: A Critical Review

**DOI:** 10.5334/cie.57

**Published:** 2023-03-15

**Authors:** M. Mahruf C. Shohel, Rasel Babu, Md. Ashrafuzzaman, Farhan Azim, Asif Bayezid

**Affiliations:** 1 University of Roehampton, London, UK; 2 McGill University, Montreal, CA; 3 Bangabandhu Sheikh Mujibur Rahman Digital University, Bangladesh, Kaliakair, Gazipur, BD; 4 University of Melbourne, Melbourne, AU; 5 University of Twente, Enschede, NL

**Keywords:** Learning Competency Framework and Approach, SWOT Matrix, Rohingya Children, Refugee, Refugee Camps, Forcibly Displaced Children, Access to Education, Basic Education, Sectarian Violence, Bangladesh, Myanmar

## Abstract

This article is based on a critical review of the Learning Competency Framework and Approach (LCFA) developed for providing education to the Rohingya refugee children living in refugee camps in Bangladesh. A sectoral approach was adopted to develop the LCFA under the leadership of United Nations Children’s Fund (UNICEF). To review the LCFA, a Strengths, Weaknesses, Opportunities and Threats (SWOT) analysis was used as an analytical tool. The SWOT analysis showed that the major strengths of the LCFA include its emphasis on pedagogical aspects, the inclusion of content on life skills, and the scope of engaging communities in the implementation phase. However, the major limitations of the LCFA comprised of lack of contents on post-traumatic mental wellbeing, child abuse, trafficking, and technology. In addition, the volume of content seemed too heavy concerning the duration of the levels. It was not clear if the LCFA was a research-based output, other than consultations. Several challenges were identified by this critical review in implementing the LCFA in the Rohingya refugee camps in Bangladesh. These include a lack of understanding of the Rohingya children’s needs, including historical, physical (both geographical and infrastructural), and livelihood, the barrier to comprehending their language and culture, and existing resource constraints for implementing this framework. Considering the Rohingya people’s perspectives, this review makes suggestions to ensure the whole education process becomes more operational, effective, successful and sustainable.

## Conflict in Rakhine State of Myanmar and the Rohingya Refugee Crisis

The ongoing global refugee crisis is one of the worst humanitarian crises in modern history. The twentieth century produced the largest number of refugees ever in recorded history (IOM, 2020). In the Asian context, the recent forced displacement of the Rohingyas from their home in Myanmar is a prime example of the refugee crisis (Shohel, 2022; Shohel et al., 2021a). According to Milton et al. (2017), the Rohingya influx into Bangladesh has been the most intense emergency that the world has encountered in the past few decades. When the Burmese King Bodawpaya conquered and annexed Arakan to the then Kingdom of Ava in central Burma in 1784, the first wave of the Rohingya refugees fled to the region of Cox’s Bazar. In the modern era, in addition to the 1942 exodus of refugees, three major influxes of the Rohingya people arrived in Bangladesh in 1977–1978, 1991–1992, and 2016–2018 to flee Myanmar Government-backed organised a genocidal and ethnic cleansing campaign (see Table 1) (Mohsin, 2020; Lewis, 2019; Reliefweb, 2019).

**Table 1:** Number of Rohingya refugees who entered Bangladesh in different times.


YEAR	NUMBER OF REFUGEES (APPROX.)	SOURCE(S)

1942	22,000	HRW (2000)

1977–78	200,000	MSF (2022); Islam et al. (2022)

1991–92	250,000	HRW (2000); Kiragu et al.(2011)

2016	80,000	HRW (2018)

2017	700,000	Reid (2021)

2018	11,432	HRW (2018)


**Figure 1:**
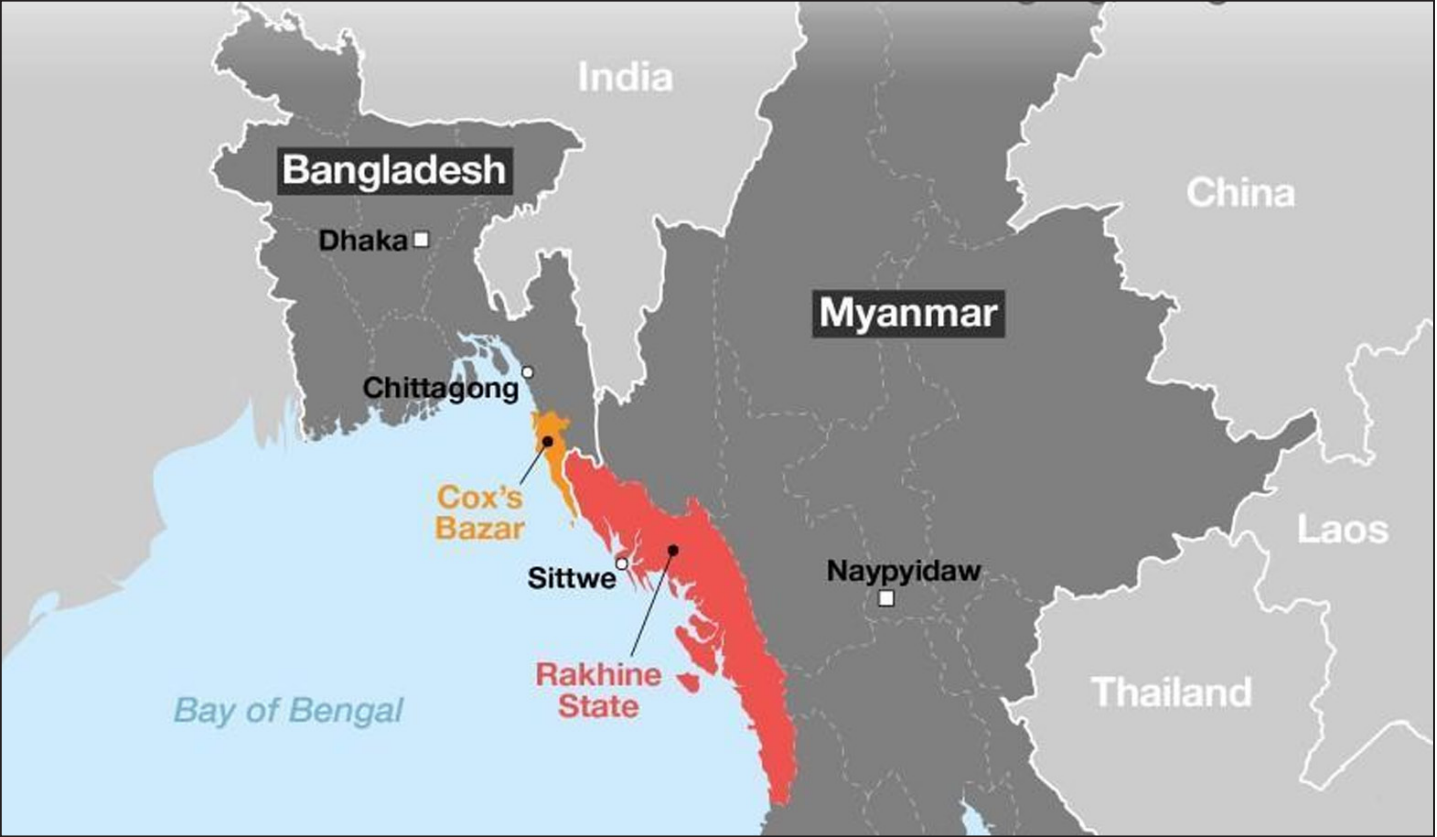
Forcibly Displaced Myanmar National to Bangladesh. *Note*: Map showing location of the Rakhine State in Myanmar and location of the Rohingya refugess in Bangladesh (DGHS, 2022), From http://dashboard.dghs.gov.bd/webportal/pages/controlroom_rohingya.php.

Despite global criticism and pressure, the Government of Myanmar has not yet followed through on the agreement of taking their citizens back, practically ignoring the fact that these people are citizens of their country (Ahsan Ullah, 2016). The United Nations (UN) termed the incident in the Rakhine State (formerly known as Arakan) as *Ethnic Cleansing* by the Government of Myanmar; subsequently, these people forcefully have taken refuge on the south-eastern coast of Bangladesh (see Figure 1) (Alam, 1999). The refugee issues are attracting international attention significantly because the policies affecting refugees are directly associated with the political and security interests of the states (Gil & Dull, 1994).

During the persecution campaign Operation Pyi Thaya in 1991, the Rohingyas initially fled to the Cox’s Bazar district of Bangladesh which is geographically the closest to Rakhine state (Marra, 2014). There are currently 34 Rohingya camps in Bangladesh in five Unions of two Upazilas (HDX, 2021). The government, non-government organisations and international agencies for humanitarian aid have been working together to provide the refugees with the necessary services during their stay in the camps (Ahmed & Ahmad, 2020). In addition, 30,079 Rohingya refugees are currently living in Bhasan Char^1^ (The Daily Star, 2022; Nguyen & Lewis, 2022).

## Education of the Rohingya Children

The Rohingya children’s access to school has been hampered since early childhood even before their mass displacement. Girls’ dropout rate was very high, and the enrolment rate was not up to the mark due to hurdles such as restricted schools, learning resources, distance of schools, lack of money, lack of skilled teachers, and safety (BROUK, 2018). Having no access to universal basic human rights, the Rohingyas and their children remained massively illiterate. In Bangladesh, NGOs work with refugees in camps and with the host communities to create Temporary Learning Centres (TLCs) and Child-Friendly Spaces (CFSs) to provide access of basic education and early childhood education (ECE) managing learning spaces with a playful environment. These learning centres provide English, Burmese, and Arabic language learning (Shohel, 2022). There was no government-approved provision of education for the Rohingya refugees in Bangladesh until 2015. With the help of the UN agencies and NGOs, the government approved and started providing nonformal basic education for the Rohingya refugees after 2015. However, there is no provision for them to continue their education beyond the basic level or to continue their education alongside Bangladeshi children in the mainstream schools.

Providing basic education is a key element for bringing necessary literacy progress and behavioural development among the Rohingya people. To address the learning challenges and gaps, a *Learning Competency Framework and Approach (LCFA)* for the Rohingya children was formulated (UNICEF, 2018). In 2019, the Ministry of Primary and Mass Education (MoPME) developed the Guideline for Informal Education Program (GIEP) for Children of Forcibly Displaced Myanmar Nationals (FDMN). This GIEP focuses on ensuring sustainable informal and fundamental education in a safe and child-friendly environment which they can use once they are repatriated. The GIEP is designed to serve as a roadmap for individuals interested in offering informal education to FDMN and their children in Cox’s Bazar, bringing some regularity to the process (MoPME, 2019).

## The Rationale of Providing Basic Education for the Rohingya Children

As per the statistics provided by the UNHCR (2018), more than 9,00,000 Rohingya refugees have arrived in Bangladesh and are dwelling in 34 camps. The male and female ratio in the migrated population is almost equal. There are approximately 5,00,000 children aged 5–18 years who are eligible for basic education but have not received it yet. Refugees aged 18–59-year-old cover approximately 40% of the population in the camps altogether (UNHCR, 2018). The Rohingya children had a very high dropout rate when attending their temporary nonformal learning centres. Only 22% of the children who entered pre-school learning centres ended up completing the nonformal primary education cycle (Feeny, 2001). Just 11% of the Rohingya refugee children were moved to secondary or post-primary education (grades 6 and 7) while 82% were enrolled in primary schools (grades 1 through 5) (UNHCR, 2018). While there are a variety of reasons for these high drop-out rates at the camp’s schools, the most prevalent factor is the economic disadvantage – the opportunity cost of attending schools. Most male students, as well as some girls, dropped out to look for jobs both inside and outside the camp because their families required income (HRW, 2019).

## Learning Competency Framework and Approach (LCFA)

The LCFA was developed under the supervision of the Education Sector^2^ based in Cox’s Bazar, Bangladesh by adopting a consultative and participatory process with multiple stakeholders and beneficiary groups. Throughout the process, the Government of Bangladesh was always kept informed and the Rohingya refugees’ views were also considered and incorporated.

As a guiding document for the education providers of the displaced Rohingya children living in the refugee camps in Bangladesh, the LCFA has provided with a blueprint for quality, protective and relevant learning. In other words, the LCFA provides a layout of what will be taught and how it will be taught through the promotion of core-level learning competencies. According to the design of the LCFA, initially, four learning levels (UNICEF, 2018) and later five learning levels were mapped to competencies against content areas (LCFA, Revised Draft, 2019):

- Level I: Equivalency: Pre-Primary (Preparatory Phase)
- Level II: Equivalency to Grade 1 and 2 Competencies
- Level III: Equivalency to Grade 3, 4 and 5 Competencies
- Level IV: Equivalency to Grade 6, 7, 8
- Level V: Equivalency to Grade 9 and 10

In phase 1, LCFA Levels 1 and 2 included contents such as Language (English and Myanmar National Language^3^), Math and Life Skills. LCFA Levels 3 and 4 included the following contents: Language (English and Myanmar Language), Math, Science and Life Skills. However, the Government of Bangladesh approved a document pertaining to Level-I and Level-II education, which is also specified in the GIEP for FDMN in Bangladesh. The curriculum matrix for Levels I and II is shown in Table 2.

**Table 2:** Learning levels: equivalence, approach, methods and material.


LEARNING LEVELS	APPROACH	DURATION	METHODS AND MATERIALS

Level I: Equivalency: Pre-Primary (Preparatory Phase)	Thematic Approach	1 Year	Child-centered, activity-based cohort of selected materials – story books, picture charts, concrete objects, play materials, art materials

Level II: Equivalency to Grade 1 and 2 Competencies	Subject Specific Approach	1 Year	Child-centered, activity based, focus on literacy and numeracy skills cohort of age and competency appropriate materials – story books, picture charts, concrete objects, play materials, art materials


MoPE, 2019, p.3 & LCFA: Revised Draft, 2019, p.7.

**Figure 2:**
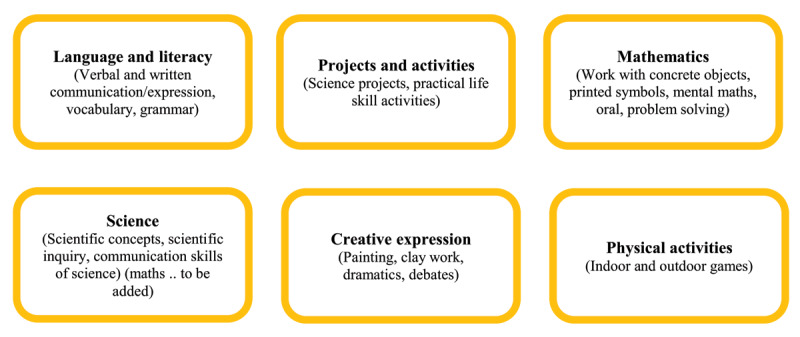
Learning Levels: Activities for Levels I and II. *Note*: Adapted from LCFA: Revised Draft, 2019, p.11.

**Figure 3:**
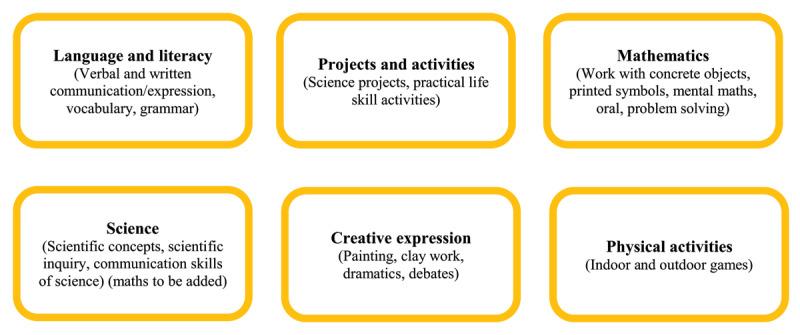
Learning Levels: Activities for Levels III and IV. *Note*: Adapted from LCFA: Revised Draft, 2019, p.11.

According to LCFA, there are numerous activities that can be assigned to children; the list and choices are virtually limitless. The following broad category is provided for the practical and realistic adoption of active learning (See Figures 2 and 3, adapted from LCFA: Revised Draft, 2019).

To implement the LCFA, UNICEF took the lead to develop a full year of teaching and learning materials (TLMs) aligned to the LCFA Levels 1 and 2, and two years of TLM aligned to Levels 3 and 4. Materials included teachers’ guides and training manuals for each subject and students’ workbooks. UNICEF collaborated with BRAC (a Bangladesh-based INGO) for technical expertise in the development, production and distribution of teaching and learning materials for Levels 1 to 4 except for the subject of English.

**Figure 4:**
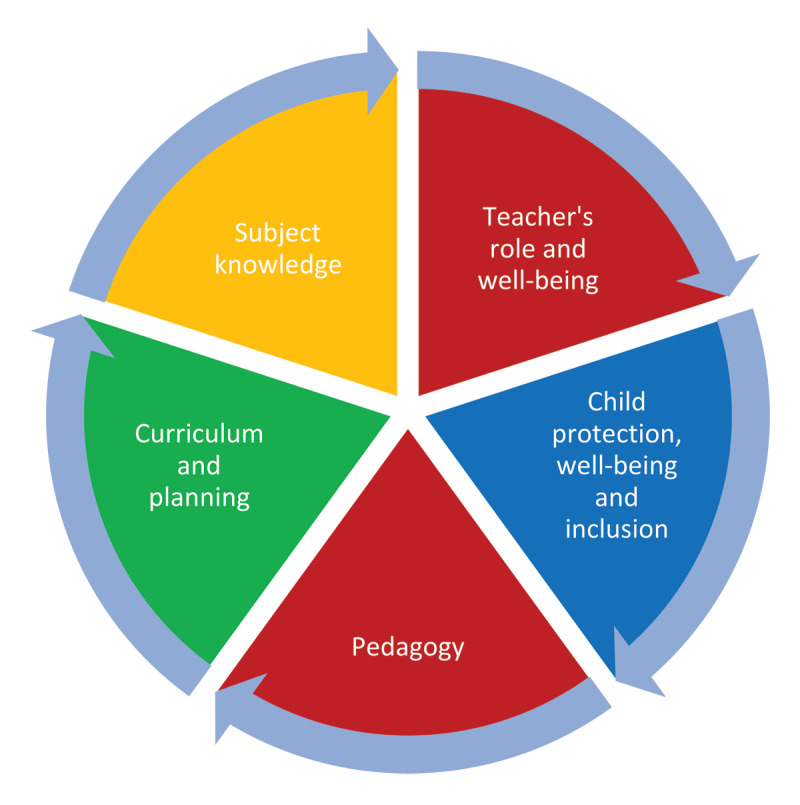
Developing Competencies among Teachers. *Note*: Adapted from LCFA: Revised Draft, 2019, p.52.

In the context of teacher development, the LCFA states that the training will primarily focus on developing competencies among instructors to successfully and efficiently execute the LCFA. The emphasis will be on subject knowledge growth, pedagogy, and centre management (Figure 4).

Teachers will be introduced to the LFCA, including its fundamental concepts, learning competencies, plus teaching and learning strategy. They will need to develop the sensitivity and abilities to implement practices that encourage no-harm, child protection, a violence-free environment, and children’s psycho-social well-being.

## Education in Emergencies: Scope of the LCFA during the Crisis

During a humanitarian response, education is viewed as the fourth priority pillar after the pillars of nourishment, shelter and health services (Midttun, 1999 & 2000; ICWAC, 2000). The uncertainty of the refugees going back to Myanmar raised concern for the education of the Rohingya refugee children living in Bangladesh. In 2017, around 453,000 children needed educational services and educational interventions in the refugee camps (HRP, 2017; ISCG, 2017; Shohel, 2022). According to government policy, humanitarian organisations have been allowed to deliver nonformal education in the makeshift settlement since 2015 (HRP, 2017). The existing education centres in the designated campsites were not capable of coping with the influx and there is no sufficient land available to establish new learning centres (ISCG, 2017). However, the existing schools for the local population, accommodating 5,000 primary and 8,000 secondary level students in Teknaf and Ukhia, were interrupted during the refugee influx as these schools were used as temporary shelters for the refugees.

Since August 25, 2017, an estimated 700,000 displaced individuals have migrated from Rakhine State to Bangladesh (UNFPA Bangladesh, 2018), bringing the total Rohingya population in Cox’s Bazar to 914,998 with 52% being female and 55% being children (UNHCR, 2022). The Government of Bangladesh (GOB), in collaboration with the UN, INGOs, and NGOs, is striving to educate these children so that they are safe, protected, and learning while staying in refugee camps (LCFA: Revised Draft, 2019). Therefore, the proposed LCFA has lots of scope for contributing to the lives of the Rohingya children through its educational interventions. This unique approach offers the opportunity to continue education up to grade 8.

## The Aim and Objectives of the Critical Review

The aim of the review was to get an insight into the LCFA and critically review and explore it to provide a comprehensive critique for improving different aspects of the LCFA. Thus, it was aiming to highlight better ways in accessing and continuing education for the displaced Rohingya children living in refugee camps in Bangladesh. In doing so, the specific objectives consisted of exploring the strengths and weaknesses of the LCFA and ways of mitigating the shortcomings of this framework and approach. The questions that were addressed in the review were as follows:

1. What are the strengths and weaknesses of the LCFA in providing access to education for the displaced Rohingya children?
2. What are the challenges of implementing the LCFA and how could those be resolved using the existing resources?
3. What could be done to provide effective and quality education for the displaced Rohingya children?

## Method

Qualitative approach was adopted to accomplish this critical review. More specifically, the authors used a document analysis approach to review the LCFA and conducted the critical analysis using a SWOT framework. SWOT analysis is a simple tool that can help faculty initiate real change in a programme and use the findings to improve the programme (Orr, 2013). That is why, the authors considered SWOT for its effectiveness as an analytical tool, often used to drive strategic planning (Creswell et al., 2000) and decision-making (Helms & Nixon, 2010; Teoli et al., 2022).

The authors are familiar with the research context and had a strong academic background with extensive educational research experience. They studied SWOT analysis as part of their academic course and used this analytical framework in their previous research (Ashrafuzzaman et al., 2010). During the analysis, the authors came up with their individual ideas and extensively discussed areas where they had differences in opinions and integrated different perspectives through a critical discussion. Rigorous arguments and thorough unpacking of thinking processes were utilised to discern concrete decisions that are free of personal biases. Multiple iterations of this process were followed for the acceptance or rejection of ideas for each of the four quadrants of the SWOT analysis.

In support of the above analysis, a comprehensive literature review was carried out using secondary sources of data from academic and grey literature (see a short review table of the key literature in Appendix 2). The findings were categorised under the four themes of the SWOT matrix.

## SWOT Matrix as an Analytical Tool for the LCFA

A SWOT matrix (Table 3) has been used to analyse the critical aspects of the LCFA. Because analysing through this matrix would not only emphasise the internal strengths and weaknesses of this approach but also the external opportunities and threats that an educational initiative might experience throughout the implementation process (Bonnici & Galea, 2014).

**Table 3:** A SWOT Matrix on LCFA.


STRENGTHS	WEAKNESSES

A. PEDAGOGY	A. CONTENTS

In the LCFA curriculum, the pedagogical approach is emphasised (LCFA: Revised Draft, 2019, p.6). The holistic development of students is highlighted. Extensive content, instructional methods, and evaluation are the focus of the LCFA (LCFA: Revised Draft, 2019). Teachers’ development and training are given top priority. LCFA emphasises a variety of teaching and learning methods, including blended learning, child-centred learning, and experience-based learning (LCFA: Revised Draft, 2019, p.6). Both formative and summative assessment methods are outlined in LCFA (LCFA: Revised Draft, 2019, p.54).	Child abuse and human trafficking are not adequately addressed. There is no content related to using contraception and family planning for upper-level students. The aspect of information and communication technology (ICT) is not included in the curriculum. There is no mention of sports in the curriculum.

**B. Life Skills**	**B. Design**

The LCFA includes life skills components for refugee children (LCFA: Revised Draft, 2019, p.10). The area of environmental awareness is addressed. The importance of children’s social cohesion is emphasized. The framework includes employability skills, music, morals, and peace.	The LCFA promotes Rohingya girls to receive a distinct shift and home-based training. During the development of the LCFA, the curriculum of other countries, notably Myanmar and the host country, was also consulted. But no study was conducted to contextualise it based on the Rakhine curriculum. There is no discussion of how the level-based skills method was verified, and there is no explanation of the basis for picking four levels (I to IV). Taking into account the time allocated for each level, the amount of content appears to be excessive. It provides a list of tasks to complete but no instructions on how to complete them (e.g., mentoring, documentation and so on).

**C. Design and Community Engagement**	

LCFA emphasises the importance of local community education. Starting of the community mobilisation process has been given the foremost priority. LCFA is an output of consultations with key stakeholders. The competency and level-based strategy are intended to promote the acquisition of learning competencies across the age groups (4 to 18 years). There are older youngsters in the camp, mainly adolescents aged 15 to 18 years, and the need of educating this group is recognised. A systematic method of situation analysis and planning will be implemented in order to fulfil the special demands of this group. The development of level-based learning competencies is in the plans. It is designed based on United Nations Educational, Scientific and Cultural Organisation (UNESCO’s) four educational pillars.	

**OPPORTUNITIES**	**THREATS**

**A. Partnership**	**A. Understanding the Children**

The government, UN agencies, and a variety of non-governmental organisations are and will be working together. MoPME is aware of the LCFA development process and willing to collaborate with other stakeholders. The framework will provide certificates and facilitate the transition of graduates into the formal system. There is provision in LCFA for a Learning Centre Management Committee, which will assist the whole implementation process. Under LCFA, different stakeholders will be able to consult on educational topics to address difficulties.	The children have been seriously traumatised. It is a difficult undertaking to educate the enormous number of affected children in the camps. Children who have had a life-changing experience require care, stability, and safety, and the need for ongoing psycho-social care and protection measures remains (LCFA: Revised Draft, p.3, 2019). Children attending the Learning Centres varied from 4 to 14 years and were categorised as never schooled, dropped out and disrupted schooling children due to displacement (LCFA: Revised Draft, p. 56, 2019). Children may have a hard time coping with the volume of content they are exposed to.

**B. Scope of Work**	**B. Cultural Barriers**

Education, safety, and psychosocial support are the key areas of LCFA, therefore, will address the holistic needs of the learners. Stakeholders like parents, government officials, and members of the community are significantly considered for implementing LCFA which will create more scope for collaboration. Mixed activities including projects, talks, and teacher-led initiatives will foster the implementation and knowledge generation process. The global competency framework is used as a reference point (LCFA: Revised Draft, 2019, p. 7), therefore there will be the possibility of developing the learners as global citizens.	In addition to knowing the learners and their culture (Rakhine), another issue is contextualising the global competency curriculum for the Rohingya children. Rohingya parents bear a gender stereotype perception. According to LCFA, safety concerns frequently reinforce conventional gender beliefs, and in this case, field-level interactions with parents revealed that traditional gender attitudes persist among community members. Boys’ educational goals are to become professionals such as doctors and teachers, whereas they are reluctant to send girls to school around the age of twelve (LCFA: Revised Draft, 2019, p. 40). If such a significant number of Rohingya migrants are granted long-term shelter in Bangladesh, the country will be subjected to a severe socioeconomic and environmental crisis. The execution of the LCFA may be interrupted due to a lack of funds.

	**C. Resource Constraints**

	There are limited teaching and learning materials available. Problems with language, teacher selection, and age differences. Lack of qualified and skilled teachers. The blended approach may be difficult for teachers to execute. Politics on a national and international level hindering resource mobilisation


## SWOT Analysis and Discussion

The concept of the LCFA appears to be a significant and positive step toward providing basic education to the displaced Rohingya children currently living in Bangladesh. Based on the LCFA, a large majority of the Rohingya children will be able to acquire their basic education, which will help them improve their learning including language, literacy and life skills.

### The Strengths of the LCFA

The major strength of the approach lies in its development process, content, pedagogic aspects and connectivity with the community. The framework was developed based on discussions with relevant stakeholders. Moreover, UNESCO’s four pillars of education- learning to know, learning to do, learning to be and learning to live together (Delors, 1996) were significantly considered while developing the LCFA. Under the theoretical base provided by Delors (1996), competencies such as environmental sensitivity, social cohesion and employability skills have been considered throughout the development process of the LCFA which would undoubtedly help the learners survive and sustain in such an emergency.

The selection of learning competencies and levels is well-balanced, allowing the displaced Rohingya children to easily complete their studies and progress from one grade to the next. In delivering this learning package in practice, it is expected that the displaced Rohingya children would be developed holistically even in this crisis period of their lives. Subjects like language, mathematics, science and life skills have been included in the approach. These would help to build a solid foundation for their further educational progression. Moreover, the inclusion of the Rohingya language would create decent educational and professional opportunities when they are back in their home country (Shohel, 2022; Sinclair, 2002).

The LCFA takes a learner-centred approach by following learning-friendly guidelines such as “keeping a safe and child-friendly space”, “doing no harm”, “maintaining gender sensitiveness according to the Rohingya culture and norms”, “inclusiveness”, and allowing “the community for active engagement” within the initiative. Much emphasis has been given in the LCFA on how the contents are delivered to the children. The multiple dimensions of the children’s lives increase the complexity of the LCFA because every child is an individual and they have their own processes of conceptual development (Bruner, 1972; Carey & Gelman, 1991; Gardner, 1991; Wellman & Gelman, 1992). Most of the Rohingya children are in acute trauma, as many of them have lost their parents in the conflict or lost close family members (Shohel et al., 2022a). In such a situation, proper care from the facilitators is most important as a healing process for their mental well-being and to construct their future. The LCFA puts emphasis on the teacher development process as well as encourages multiple ways of teaching and learning (MWTL) based on multiple intelligence theory (Davis et al., 2012). The pedagogical approach provided in the LCFA appears to be adaptable and achievable, giving the student plenty of room and time to manoeuvre comfortably while receiving a quality education.

Formative assessment encourages learning by providing immediate feedback on students’ performance which eventually helps to make a successful summative evaluation (Jones, 2005). The provision of formative and summative assessments would help strengthen children’s learning and engagement with the learning activities. Therefore, this approach is strong from a pedagogical perspective as well.

Community involvement in the implementation of the framework is a good example of a collaboration model for implementing curricula (Hornby, 2011). Literature shows that parental involvement in the educational process has some extraordinary benefits including an increase in attendance and learning achievement (Erdoğan & Demirkasımoğlu, 2010; Desimone, 1999; Erdener, 2016; Erdener & Knoeppel, 2018). Data shows that 57% of girls and 60% of boys among 6–14 years old Rohingya children have attended learning centres since arriving in Bangladesh. However, at the ECCD stage (ages 3–5), attendance was shockingly poor, at just 43%. Furthermore, adolescent girls had even lower attendance, just 4%, compared to 14% for adolescent boys (Education Sector, 2018). Thus, the inclusion of parents or guardians of the children might improve their attendance in the centres and achievement of their intended competencies by them.

The LCFA proposition aims to teach the Rohingya children with the help of facilitators from the host community and the Rohingya ethnic people. This is a very realistic and appropriate approach to selecting facilitators to teach or facilitate the Rohingya children with basic schooling. A combination of local and the Rohingya teachers is expected to be more effective in terms of providing more quality education to the Rohingya children since they have an adequate understanding of the traditional Rohingya context, culture and society.

The overall aims, guiding principles, pedagogical approaches, and implementation planning of the LCFA proposition look systemic and well-planned. However, active and meaningful participation by all the stakeholders in the implementation phase would lead the project toward success.

### The Weaknesses of the LCFA

It is questioned how far the traumatised children would be able to acquire the education outlined in the LCFA if they lived in a crowded refugee camp. Issues like child exploitation, human trafficking, child psychology, needs of the Rohingya children and gender violence received much less attention than they deserve. There is a lack of a secure and safe environment which has been identified as one of the major educational challenges in any refugee camp (Bonyan Organization, 2022). Therefore, education should provide awareness as well as the potential to prevent these harmful malpractices, particularly during humanitarian crises. About sexual abuse, a section on “good touch and bad touch” has been included, but there is much more to consider. The Rohingya women and girls in Cox’s Bazar are particularly vulnerable due to their gender, refugee status, and ethnic affiliation (Nordly, 2018; Plan International, 2018). Psychosocial counselling and effective content delivery, education can play an important role in their recovery. However, there is a shortage of adequately trained teachers in the refugee camp to provide opportunities of learning and mental support for the learners with severe trauma, mental disorder and stress. Many children have physical disabilities (Bonyan Organization, 2022) which could be a potential challenge in implementing the LCFA.

One of the most challenging tasks was to provide health facilities to the Rohingya pregnant women as well as babies (Shatil et al., 2017). Therefore, through education, the concept of health care, mental well-being and family planning could be provided to the upper grades and adolescent boys and girls so that they have a clearer idea of how to take care of themselves.

In this era of technological innovation and digitalisation, the Rohingya children should not be deprived of the benefit of gaining ICT skills and digital literacies as well as having opportunities to use them in their learning. The LCFA does not contain any scope for using ICT in teaching and learning. Therefore, if possible, the use of ICT and emerging technologies should be included so that they can get access to blended learning and Online Distance Teaching and Learning (ODTL) opportunities (Shohel et al., 2022b). Remote learning through technologies is also known as distance education or e-learning (Parsad & Lewis, 2008), mainly a technology-based method (Shohel et al., 2022c; Shohel et al., 2021c), which is getting popular with emerging technologies for its flexibility and low cost (Balalle & Weerasinghe, 2021; Bartley & Golek, 2004). Moreover, ODTL allows students to play a more active role in their education and they can learn at anytime from anywhere (Shohel et al., 2021b).

Another shortcoming related to content is that the curriculum of the LCFA hardly creates scope for play-based learning and sports. However, play and sports have a significant role in improving learners’ physical and mental health (Bailey, 2006). Recently, a football match was arranged for the Rohingya children with a view to promoting psychological development, physical health and social well-being (IOM, 2020). Such opportunities need to be expanded beyond one-off events and the opportunity for sports and physical activities should be included in the curriculum. A potential infrastructural challenge is there regarding the place to play. Literature shows that in the camp there is obviously a scarcity of educational buildings or a safe study place for the child (Bonyan Organization, 2022).

One of the major challenges in dealing with refugee children’s education is that there is hardly any information regarding their previous education (World Economic Forum, 2016). Therefore, in such a situation it was very essential to collect and analyse their educational information as much as possible and to contextualise the framework accordingly. Moreover, how the four levels (I – IV) were identified and on what basis, was not clarified in the approach. It has been said that the level-based competencies approach has been tested, but how it was done was not mentioned in the document. Without clarifying these, it is difficult to establish the reliability and practicality of this framework. In general, it is observed that the LCFA has presented a number of tasks to accomplish but how these would be done is rarely discussed. For example, tasks related to mentoring and documentation were mentioned, but the process of conducting these was not discussed. The inclusion of these would enhance the rigour of the model.

The LCFA promotes separate shifts based on gender and provisions for home-based training. This may be well received by the Rohingya families because women and girls have additional needs due to social norms and cultural practices. Thus, parents or guardians are less willing to send their daughters to schools without gender-separate classrooms (Ripoll et al., 2017). The Rohingya girls too had concerns when they did not have a gender-segregated classroom because they felt uncomfortable (Education Sector, 2018). However, a gender-segregated classroom, in the long run, could disrupt inclusion and encourage the isolation of refugee girls in mainstream society.

Finally, the LCFA proposal does not specify how many children will be able to get an education through this educational initiative at any given phase or how long this LCFA will be implemented for the Rohingya refugee children. So, it is difficult to foresee the ultimate outcomes of the learning competency framework and approach.

### The Opportunities of the LCFA

The LCFA has two major advantages for effective implementation: a substantial collaboration with various potential organisations and a wide variety of innovation possibilities. The Governemnt of Bangladesh, specifically the Ministry of Primary and Mass Education (MoPME), UN bodies, and local and international NGOs were all informed and involved in the development of the system. As a result, there is a lot of space for future collaboration with these organisations. By fostering a more constructive heterogeneous relationship (Webb, 1982) and recognising the differences, proper teamwork increases the likelihood of a project’s success and leads to its smooth operation (Swing & Peterson, 1982). So, a result-oriented and professional partnership with these bodies would ease the way to reach the goal of the LCFA. The framework has the ambition to transfer the graduates to the formal education system once they complete the course. This assimilation into formal education is another opportunity for the Rohingya children to get into the mainstream. According to the LCFA, there will be a learning centre management committee. If this committee involves the local inhabitants, it will provide ownership to the community through this model. Thus, the school management committee would be more caring and active regarding the implementation of the framework. Epstein (2005) also argued that parental involvement functioned effectively for school improvement by enhancing the curriculum, instruction, assessments, and different aspects of school management.

The LCFA invites a variety of methods and activities to be examined throughout the implementation process. These would create scopes for innovation regarding educational interventions for refugee children. Moreover, an alignment between the global competency framework and the LCFA will create an opportunity to universalise the approach. Therefore, even being a child of a refugee camp, they would be able to compete globally in their learning achievement and progression.

### The Threats and Challenges of the LCFA

There are a few potential threats to adopting the framework, such as teachers’ lack of understanding of the contents and complex attributes of the Rohingya children, resource constraints, cultural obstacles, and the effect of local, national, and international politics. Moreover, because of the conflict and the displacement from their home, they arrived with limited and interrupted education (Dooley, 2009; Dryden-Peterson, 2016). As a result, individuals also tend to have learning gaps. Furthermore, according to the LCFA, there would be a wide range of differences among learners in terms of age, abilities, and degrees of educational attainment. As a result, correctly understanding them and designing instructions for them would be a difficult task. Understanding their language would be another potential challenge for these teachers if they are not recruited from Rohingya refugees. As similarities exist between the Rohingya language and Chittagonian^4^ language (Hoque, 2015), recruiting teachers from that region might be a better solution for this aspect.

Not only understanding the learners and their culture but also contextualising the global competency curriculum for the Rohingya children is another crucial challenge. If this is not done properly, the children would not find the lessons exciting and would not engage themselves in the teaching and learning activities. These would not be helpful for them when they are sent back to their homeland. The most potential risk might be the resource constraint because providing education in such an emergency where the uncertainty of getting back to normal life for the refugees demands more financial and supplementary resources. Developing different teaching and learning materials is a challenging and time-consuming task. The scarcity of these has already been identified as a potential barrier for providing education in the refugee camps (Education Sector, 2018). However, this framework should encourage a blended learning approach (Cleveland-Innes & Wilton, 2018; Shohel et al., 2022c; Shohel et al., 2021d) to support the learning journey of these children.

Having motivated and skilled teachers to teach such a special group of displaced children might be another potential challenge. To overcome these challenges, teachers would have to play a vital role, and there should be close supervision and continuous professional development for these teachers.

The Rohingya refugee community desires a new curriculum for their future generation (Reidy, 2020). The way LCFA is designed might create acute pressure on the children. As the contents of three grades would be taught in two years, or eight years’ content in four years, the children might have to face additional pressure on them. Therefore, a balance would have to be made therein to address individuals’ needs while delivering the contents. There is no indication of criteria or assessment process of students to select them for levels I, II, or III or other levels in the LCFA framework. There should be certain specified criteria to select children in different levels. Moreover, refugee children would not be able to get admission into mainstream schools unless they are registered. However, it is not clear in the framework, how this grade eight level competencies would be equivalent to the Myanmar curriculum. Therefore, the burning question is whether or not this will be accepted in Myanmar? This essential question needs to be answered by negotiating with the Government of Myanmar, which is an important area to work on. If these are not properly sorted out beforehand, then the framework would not reach its ultimate goal of providing access and continuity in education that empowers the disadvantaged Rohingya children.

National and international politics on the Rohingya crisis are some potential concerns for the implementation of this framework. Planning a four-year educational intervention for them might raise the question regarding the true intention of the initiative because offering a four-year intervention means those who are behind this framework might expect that the Rohingya children would be in the refugee camp at least for the next four years. Thus, those who do not appreciate the long-term existence of the Rohingya refugees in Bangladesh might interpret this framework as an intentional measure to ensure the long-term accommodation of the Rohingya children in Bangladesh. Therefore, a pertinent question now is how long the Rohingya would live in Bangladesh and to what extent educational interventions should be offered to them and how educational opportunities could be offered to them.

Nevertheless, without clarifying these basic questions, it is difficult to go into long-term planning and develop educational initiatives. So, unless the right decision comes, implementing this framework might pose a risk of heightening tension between the host and refugee communities.

### Suggestions for Further Improvement

The entire LCFA proposition is based on the ideas of multiple local, and international agencies and organisations as an outsider approach for bringing literacy and educational development among the displaced Rohingya people’s children. To enhance the LCFA proposition, the Rohingya people’s perspectives and suggestions have to be considered so that the whole education process becomes more effective, engaged, operational, fruitful and sustainable. According to Islam et al. (2019), as children showed decreased interest in learning life skills and mathematics, the curriculum was revised, and hands-on activities were incorporated into this educational initiative.

Children from the Rohingya minority have an uncertain future, which causes frustration and despair. They are more vulnerable to human trafficking, child marriage, exploitation, and abuse if they do not have proper schooling opportunities. Young girls and boys can benefit from skills training because it empowers them, boosts their confidence, and provides them with the abilities they will need in the future. With the correct investment in education, the Rohingya youngsters would be able to begin creating their own destinies and contribute more to their communities.

According to Education Cluster, REACH & UNICEF (2021), while the introduction of the LCFA in camps got positive comments from most teachers, it apparently reflected certain existing inequalities between children of different ages and genders. To minimise inequalities, the LCFA should concentrate more on these issues. On the other hand, because of the shutdown of the learning centres due to COVID-19, there was a further delay in implementing the LCFA to provide Myanmar equivalent formal education for the Rohingya refugee children.

## Conclusion

The Rohingyas are ill-fated people who have been deprived of their civic rights in Myanmar for many decades. The Government of Bangladesh has taken initiatives to arrange for their rehabilitation, after their forced migration to Bangladesh. Moreover, refugees are always in a disadvantaged situation when it comes to access to education (Shohel et al., 2022a). Their education falls under the umbrella of education in emergencies (Shohel, 2022). As a result, they need to acquire specially and carefully designed educational competencies. Despite its weaknesses, limitations, and challenges, the LCFA offers potential means to improve the pedagogical approaches and learning contents at different levels to Rohingya refugee children who have been denied their right to education. However, recently it has been agreed to piloting Myanmar school curriculum to provide education to limited number of the Rohingya children. So far, text books were printed and distributed for pre-primary, grades 1 and 2. Supplementary materials were supplied to bridge the gaps for grades 1 and 2 to ensure a smooth transition from the LCFA to Myanmar school curriculum (Education Sector, 2022).

Considering the Rohingya refugees’ fluid and chaotic life in camps, a set of cross-sectoral interventions including access to education and life skills training programmes is crucial to decreasing the risks of diseases, trafficking, drug misuse, early marriage, and exploitative harmful and predatory jobs. Additionally, considering the gender norms and gender violence, it is necessary to ensure that girls and young women can participate in education through the proposed learning competency framework process as learners and teachers through targeted approaches (Talbot, 2013). Within this framework, these Rohingya refugee children could be educated with fundamental health education as well as trained with skills to engage in income generation activities so that they can earn their livelihood. By overcoming the shortfalls outlined above, the framework should be able to engage the Rohingya children in a constructive educational process. As an outcome, they will be able to transcend their challenges, such as traumatic events and the loss of loved ones, as well as the hardships of living in camps or temporary shelters, by taking advantage of the opportunities provided for them to reach their full potential as human beings. Progression in education will create hope for them. After returning to Myanmar, they will be easily rehabilitated and become future contributors to their community and national economy.

## Additional Files

The additional files for this article can be found as follows:

10.5334/cie.57.s1 Appendix A. List of acronyms used in this article.

10.5334/cie.57.s2 Appendix B. Short review table of the key literature used.

## Footnotes

A small island of Bangladesh located in the Bay of Bengal. It is 37 miles away from the mainland Bangladesh and currently hosting Rohingya refugees.

Education Sector is the umbrella term for the joint efforts of NGOs working together in the field of education. It is widely used by the development organisations (both national and internation) working in Cox’s Bazar.

There are many languages spoken in Myanmar. But Myanmar national language is Burmese. In here, Myanmar Language refers to Burmese.

A dialect that is used widely in the Chittagong division of Bangladesh.
