# Supplementary material for: Learning Competency Framework and Approach for the Displaced Rohingya Children Living in Bangladesh: A Critical Review
Source: Contin Educ. 2023 Mar 15;4(1):50–66. doi: 10.5334/cie.57 (PMC11104321; doi:10.5334/cie.57)
Supplement: Appendix A. — List of acronyms used in this article. [file cie-4-1-57-s1.pdf]

Appendix A: List of acronyms used

Acronym	Full form
BEP	BRAC Education Programme
BRAC	A Non-Governmental Organization, originally founded in 1972 as the Bangladesh Rehabilitation Assistance Committee and later known as the Bangladesh Rural Advancement Committee, now branded as BRAC.
CFS	Child-Friendly Space
ECCD	Early Childhood Care and Development
ECD	Early Childhood Development
FIVDB	Friends in Village Development Bangladesh
GoB	Government of Bangladesh
HDX	Humanitarian Data Exchange
HRP	Humanitarian Response Plan
ICT	Information and Communication Technology
IED	Institute of Educational Development
ICWAC	International Conference on War-Affected Children
INGO	International Non-Government Organisation
IOM	International Organisation of Migration
ISCG	Inter-Sector Coordination Group
LCFA	Learning Competencies and Framework Approach
MI	Multiple Intelligence
MoPME	Ministry of Primary and Mass Education
MSF	Médecins Sans Frontières
MWTLI	Multiple Ways of Teaching and Learning
NGO	Non-Government Organization
REACH	Research, Evaluation and Analysis in Crisis for Humanity
SWOT	Strengths, Weaknesses, Opportunities and Threats
TLC	Temporary Learning Centre
TLM	Teaching and Learning Materials
UN	United Nations
US	United States
USA	United States of America
UNESCO	United Nations Educational, Scientific and Cultural Organisation
UNHCR	United Nations High Commission for Refugees

UNICEF	United Nations Children's Fund
