# Supplementary material for: Learning Competency Framework and Approach for the Displaced Rohingya Children Living in Bangladesh: A Critical Review
Source: Contin Educ. 2023 Mar 15;4(1):50–66. doi: 10.5334/cie.57 (PMC11104321; doi:10.5334/cie.57)
Supplement: Appendix B. — Short review table of the key literature used. [file cie-4-1-57-s2.pdf]

Appendix B: Short review table of the key literature used

Author(s)	Published from	Purpose	Type of Source	Summary Points
American Immigration Council (2021)	USA	Clarification of definitions, regulations and boundaries of the US Refugee Law and Policy	Report	The US government considers forceful displacement a crucial problem that they do not intend to keep unaddressed. Their definitions, means and procedures are valuable evidence for this paper.
Burmese Rohingya Organisation UK (BROUK) (2018)	UK	Problematising the Right to Education among the Rohingya Refugees and generating recommendations	Report	This report identifies the adequacy of resources (teachers, infrastructure), social acceptability (religious, community acceptance) and accessibility as the key obstacles in the Right to Education trajectory of the fled Rohingya refugees. As recommendations, the paper highlights mainstreaming the Rohingya people in terms of permission for formal education, investment and empirical research.
Druce (2020)	Malaysia	Contribute to the understanding of the conflicting factors of the state with the Rohingya people, compatibility and management issues.	Book chapter	The chapter elaborates on the ideological clashes in the Buddhist-Muslim coexistence in the Rakhine state and the effect of a long history of colonisation. The latter section critically examines the aftermath of post-independence 'Burmanisation' and the state-wide social injustice and torture.
Dryden-Peterson (2016)	UK	Developing an understanding through a comparative enquiry of how the right to education for all can create equal participation in society.	Article	Although the comparison set on refugee education from WWII till date, this article sheds light on the global premises of refugee education as a fundamental human right and its everyday practices. The paper brings in a debate on segregation between discourses, norms, doctrines and mechanisms of different nation states, which duly help us frame the obstacles of mainstreaming refugees into society.
Education Sector (2018)	Bangladesh	An in-depth assessment of Education in Emergency in the Cox's Bazar context better understands attendance, enrolment and obstacles.	Report	After critically examining the existing access of the displaced Rohingya children, this report projects recommendations on accepting more alternative means to broadening access and opportunities to education, engaging more effective teaching-learning methods and resources, and deploying assessment and monitoring mechanisms.
Human Rights Watch (2019)	Bangladesh	Reporting on how education as a fundamental human right is overlooked in the lives of the Rohingyas as citizens and refugees.	Report	This publication reports evidence on how the Rohingya refugee children have been deprived of accessing education as a fundamental human right. The discourse presented in the paper helps us strengthen our debate in support of ensuring primary education for them.
Isaacs (2016)	UK	Bringing up first-hand evidence of how Rohingya refugees lack access to primary quality education along with other social needs.	Blog series	The blog, through presenting heart-aching proofs of the barely human conditions of the Rohingya refugees, pulls the discussion on the dilemma of taking constructive policy decisions regarding their education through a singular Bangladeshi or Burmese perception of the displaced. The debate gives birth to the need to prioritise the idea of acquiring the Rohingya people as themselves.

LCFA: Revised Draft (2019)	Bangladesh	Learning Competency Framework and Approach for Children of Displaced People from Rakhine State, Myanmar in Bangladesh	Curriculum framework	The essential reference document for this research.
The Daily E-Kaler Kontho (2018, November 16)	Bangladesh	A critical review of the Bangladeshi government's diplomatic briefing on repatriation of the displaced people from Rakhine	Newspaper article	This news article represents one of the many features of the Rohingya influx issue that debates the Bangladeshi government's intention for the successful repatriation of Rohingya refugees. This article in the mainstream print media creates a scope of critically rethinking and assessing the educational initiatives taken by the government for the displaced.
UNFPA Bangladesh (2018)	Bangladesh	A comparative review of the Rohingya influx to predict the future	News article	This article presents past initiatives, including educational development. The report effectively attempts to calculate the aftermaths and probable future endeavours with the ill-fated people utterly dependent on the mercy of different authorities.
World Economic Forum (2016)	USA	Problematising refugee education and proposing solutions	Article	The article looks at the educational arrangement for refugees, specifically for the Rohingya people, as a pressing issue and proposes evidence-based propositions to tackle curricular and administrative crises in the scenario.
